# Bathymetric Monitoring of Alluvial River Bottom Changes for Purposes of Stability of Water Power Plant Structure with a New Methodology for River Bottom Hazard Mapping (Wloclawek, Poland)

**DOI:** 10.3390/s20175004

**Published:** 2020-09-03

**Authors:** Dariusz Popielarczyk, Marian Marschalko, Tomasz Templin, Dominik Niemiec, Isik Yilmaz, Barbara Matuszková

**Affiliations:** 1 Department of Geodesy, Faculty of Geoengineering, University of Warmia and Mazury, Heweliusza 5, 10-724 Olsztyn, Poland; tomasz.templin@uwm.edu.pl; 2 Department of Geological Engineering, Faculty of Mining and Geology, VŠB-Technical University of Ostrava, 17 listopadu 15, 708 33 Ostrava, Czech Republic; marian.marschalko@gmail.com (M.M.); dominik.niemiec@vsb.cz (D.N.); barbara.matuszkova@vsb.cz (B.M.); 3 Department of Geological Engineering, Faculty of Engineering, Cumhuriyet University, Sivas 58140, Turkey; isik.yilmaz@gmail.com

**Keywords:** bathymetric monitoring, alluvial river bottom changes, stability, water power plant structure, GNSS/SBES measurements, river bottom hazard mapping, Wisła River, Poland

## Abstract

The aim of this research was to produce a new methodology for a special river bottom hazard mapping for the stability purposes of the biggest Polish water power plant: Włocławek. During the operation period of the water power plant, an engineering-geological issue in the form of pothole formation on the Wisła River bed in the gravel-sand alluvium was observed. This was caused by increased fluvial erosion resulting from a reduced water level behind the power plant, along with frequent changes in the water flow rates and water levels caused by the varying technological and economic operation needs of the power plant. Data for the research were obtained by way of a 4-year geodetic/bathymetric monitoring of the river bed implemented using integrated GNSS (Global Navigation Satellite System), RTS (Robotized Total Station) and SBES (Single Beam Echo Sounder) methods. The result is a customized river bottom hazard map which takes into account a high, medium, and low risk levels of the potholes for the water power plant structure. This map was used to redevelop the river bed by filling. The findings show that high hazard is related to 5% of potholes (capacity of 4308 m^3^), medium with 38% of potholes (capacity of 36,455 m^3^), and low hazard with 57% of potholes (capacity of 54,396 m^3^). Since the construction of the dam, changes due to erosion identified by the monitoring have concerned approximately 405,252 m^3^ of the bottom, which corresponds to 130 Olympic-size pools. This implies enormous changes, while a possible solution could be the construction of additional cascades on the Wisła River.

## 1. Introduction

This engineering-geological study was motivated by the problem of potholes (local morphological depressions) in the bottom of the Wisła River in the gravel-sand alluvium behind the dam of the biggest Polish water power plant Włocławek. The formation of potholes endangers the concrete structures of the water power plants and the threshold which serves for partial water flow stabilization.

**Figure 1:**
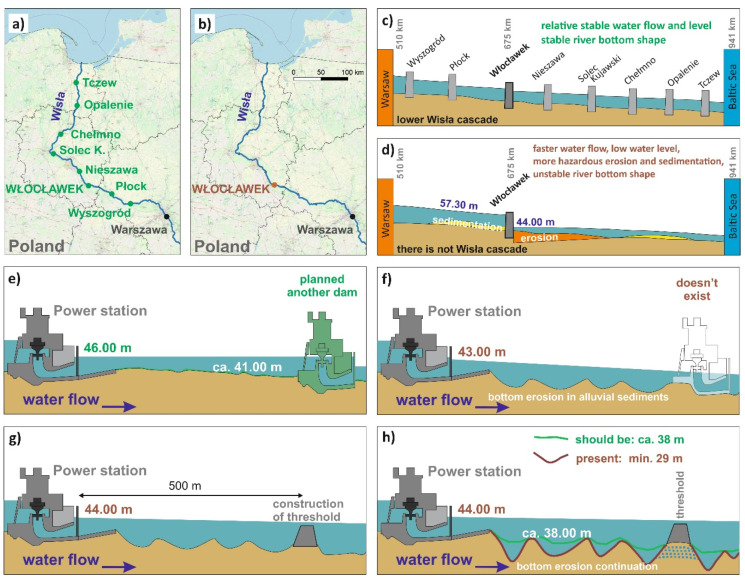
Cause of the problem on power plant Włocławek—motivation for research engineering-geological case of study: (**a**) Planned cascade of dams with optimal regime of sedimentation and erosion of the river bottom. (**b**) Realized dams with current problems of erosion of river bottom (only one power plant dam was constructed). (**c**) Planned dams in 1956. (**d**) Only one dam built in 1970. (**e**) Planned conditions. (**f**) Existing conditions. (**g**) Improved conditions as for river bottom erosion due to a threshold. (**h**) Erosion of the bottom continues and the threshold is endangered.

The Polish government originally planned to build eight water power plants north of Warszawa on the biggest Polish river, the Wisła River (Figure 1a), which would optimize the hydraulic conditions of the river in relation to the ongoing erosion and sedimentation processes in the river. The construction of dams may have a varying influence on the geological environment of river courses [1,2,3,4]. Due to financial constraints, only one water power plant was built in 1970: Włocławek water power plant (Figure 1b). The originally planned hydrological conditions arising from the set of eight dams is shown in Figure 1c. The absence of the seven dams has caused extreme hydrological conditions, with a significant drop in the river level (Figure 1d), leading to significant erosion processes behind the dam. Figure 1e shows what the originally planned conditions would be, while Figure 1f shows the unfavourable hydrological conditions as the construction of only one water power plant caused more significant erosion and formation of a high number of potholes. As a result, a protective threshold was constructed on the river, which is supposed to improve the conditions (Figure 1g). The conditions improved partially, but it is evident that the forming potholes endanger the threshold stability (Figure 1h) [5,6,7,8].

**Figure 2:**
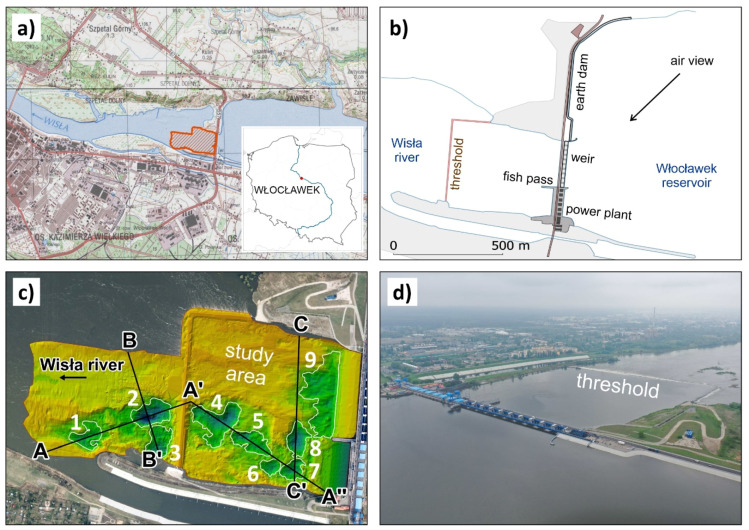
Study area: (**a**) location of power station Włocławek, (**b**) power station construction, (**c**) cross sections of the measured potholes, (**d**) photo-documentation of the water power station.

The specific conditions of the engineering-geological case study are related to the research in the study area (Figure 2) of alluvial gravel-sand environment of the Wisła River behind the Włocławek water power plant dam. This stretch of the river suffers from intense river bed erosion, which causes extensive geomorphological changes resulting in a high number of potholes in this geological environment. The morphology of river channels is very important from the point of view of the fluvial process character and formation of potholes [9,10,11]. One of the factors that influences the formation of potholes is the drop in the Wisła River water level behind the dam, which amounts to 13 m. Another factor is significant changes in the water flow and water level, due to the varying requirements for the technical-economic operation of the water power plant. Particularly, this concerns the requirements related to peaks. Another factor is changes in the water regime caused by extraordinary processes, such as floods.

**Figure 3:**
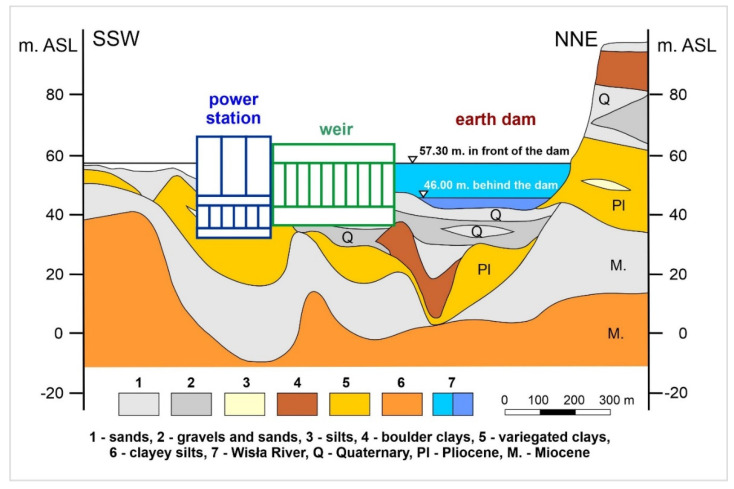
Geological cross section of the Włocławek water power plant.

As reported in Hazell et al. [12], Delgado et al. [13], Rommens et al. [14]; Kiracofe et al. [15], and Marschalko et al. [16], the character of the rock material influences the geological structure of the alluvium, as well as the physical-mechanical parameters, enabling the planning activities in the geological environment. The foundation conditions of the Włocławek water power plant can be seen in Figure 3, while the most important section with the most prominent interaction is bound onto the Quaternary gravel and sand. The next section is characterized by Pliocene clay. In the bedrock, there are Miocene sand and clayey silts. Geological structure bedrock is one of the most important factors in the river activity. For this reason, it is decisive whether the bedrock is made of rocks or soils as it conditions the resistance of the rock massif [17,18,19]. In light of the above, the Wisła River bedrock is made up of low-resistant soils.

So far, the risk of potholes formation has been assessed by means of classic geodetic measurements (theodolite) and analogue depth measurements (manual probe/lead line). These measurements were taken locally, only on a few profiles, without giving full information about the size and shape of the bottom and potholes. Classical survey methods do not provide with data for reliable monitoring. It was difficult to apply effective methods to eliminate the risk of dam structure damage.

The proposed methodology covers the whole process of testing the safety of power plant structures: planning/conducting an integrated high-accuracy bathymetric GNSS and hydroacoustic measurements, developing the data, analysing it, selecting criteria, assessing risks, and proposing appropriate solutions.

The purpose of this study was to evaluate the erosion changes of the river bed and their effect on the power plant operation. Despite the fact that a threshold was built to stabilize the water flow in the river, the study is also important because the potholes in its vicinity influence the stability of the threshold. This study also aims to propose a remediation project to backfill the potholes. This may lead to the stabilization of the river bed and the surrounding structures of the water power plant.

The final result of this work is a customized river bottom hazard map, which takes into account a high, medium, and low risk levels of the potholes for the water power plant structure. The research method was a 4-year-long geodetic/bathymetric monitoring of the river bed carried out using integrated methods of GNSS (Global Navigation Satellite System), RTS (Robotized Total Station), and SBES (Single Beam Echo Sounder). Next, with the help of the new methodology, we produced a special, customized river bottom hazard map. The production of the map allowed to create a technical remediation proposal for holes backfilling and to propose a future monitoring system for the river bed, including its frequency, for the sake of sustainable operation of the Włocławek water power plant.

## 2. Bathymetric Monitoring

**Figure 4:**
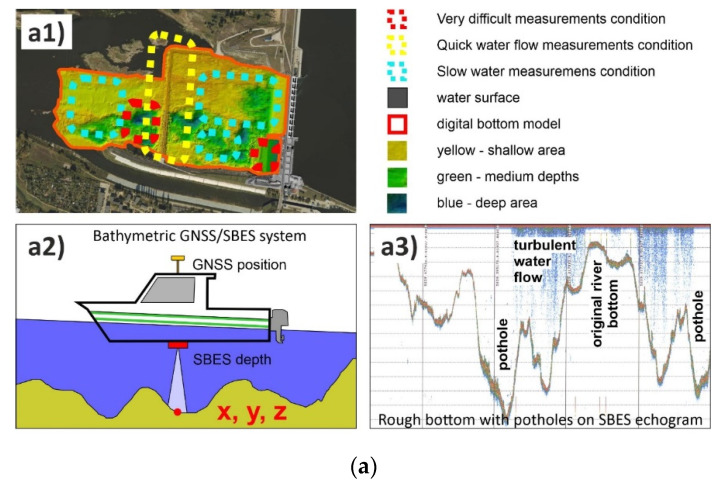

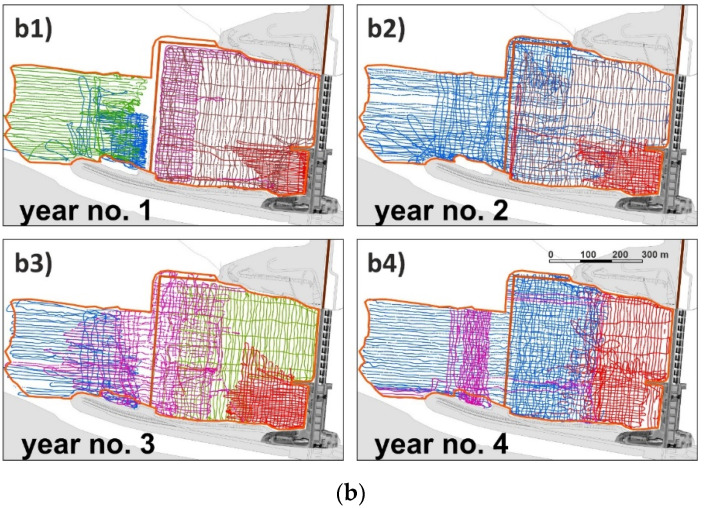
Bathymetric monitoring (**a**) measurements condition, (**b**) stages of measurements, (**a1**) study area measurements condition, (**a2**) bathymetric equipment, (**a3**) rough bottom and turbulent water flow, (**b1**–**b4**) hydrographic motorboat trajectories during measurement stages.

To analyse the erosion and sedimentation process of the Wisła River bottom below the Włocławek power plant (Figure 4(a1)), 4-year long geodetic/bathymetric measurements were conducted. In general, bathymetry shows a 3D model of the underwater bottom shape. This allows analysing changes in the shape of the bottom erosion and movements of sedimentation. Currently, research on the aquatic environment is carried out using modern geodetic and hydroacoustic measurement techniques, supported by remote sensors and processing algorithms [20,21,22,23]. An integrated GNSS satellite positioning, Robotized Total Station (RTS) and a Single Beam Echosounder System (SBES) were used for hydroacoustic depth measurements (Table 1). These bathymetric surveys were made in real time using a small motor boat (Figure 4(a2)).

The key problem during practical surveys (Figure 4(a3)) were the height of the water surface and waterflow speed during depth measurements (rough and choppy water and quick water level changes depending on the schedule of the power plant work and on the distance from the dam). Particularly dangerous were the areas behind the turbines of the power plant and behind the damming threshold. Zones of different measurement conditions are presented in Figure 4(a1).

Measurements should be carried out on the basis of designed measurement profiles (the same each year). In fact, the routes differed slightly from the planned lines due to navigation errors, difficult measurement conditions and power plant operation schedule. That is why the GNSS/RTS/SBES bathymetric measurements were carried out in several stages in each of the four subsequent years of river bed inventory (Figure 4(b1–b4)).

All depth measurements should be related to the common water level (reduced to the common vertical datum). In inland bathymetry, this physical water surface is usually stable and can only change slightly in time (do not need any reductions; easy to elaborate raw data). An exception to the rule is reservoirs of hydroelectric power stations, where the water level could change significantly over time and distance. The example is the biggest flowing power station in Poland: Włocławek hydroelectric power plant which causes up to 2 m vertical water level movement during the day.

Bathymetric data were acquired using single beam echosounder which needs vertical motion corrections (heave). Additionally, all raw depth data should be referenced to the common water level.

To reach the final reduced depth measurements, it is necessary to obtain the precise vertical position of the platform. The classical geodetic Total Station, GPS RTK (Real Time Kinematic), GNSS OTF (on the fly), and readings from local water gauges were used for sub-centimetre estimation of water level changes during hydroacoustic measurements. The finally reduced bathymetric data were used to elaborate 3D bottom elevation models for further erosion and sediment analysis.

## 3. Evaluation of River Bed Changes

**Figure 5:**
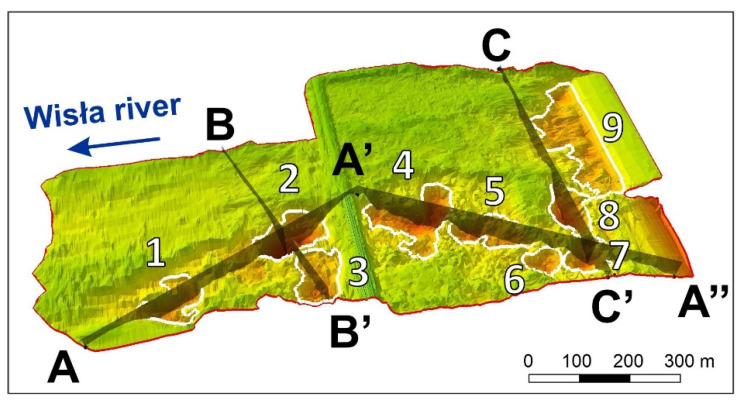
Study area. Potholes and cross sections.

The area of interest was spatially evaluated based on basic criteria: selected model cross-sections (AA’, A’A”, BB’, CC’ - to represent the vertical morphological changes in the river bed) and the changes in the cubic capacity of the Wisła River bed erosion, generally, on the whole study area, and additionally in the biggest potholes (1–9), selected for analysis (Figure 5).

### 3.1. Cross-Section Evaluation

**Figure 6:**
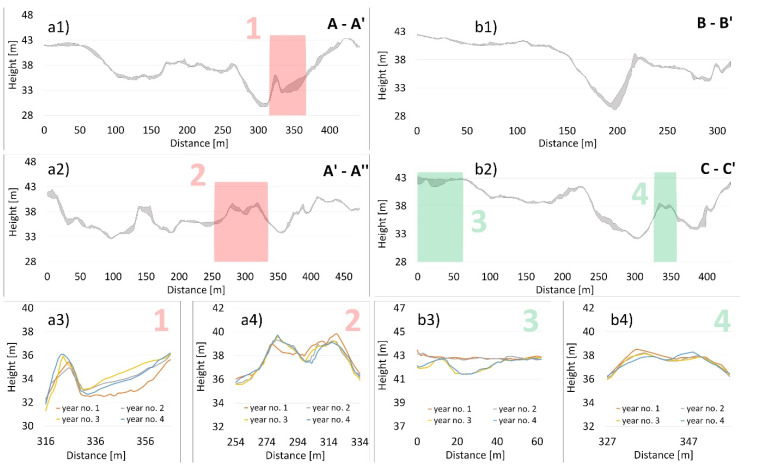
Changes in the river bed in (**a1**) cross-section A-A′, (**a2**) cross-section A′-A′′, (**a3**) detail No. 1, (**a4**) detail No. 2, (**b1**) cross-section B-B′, (**b2**) cross-section C-C′, (**b3**) detail No. 3, (**b4**) detail No. 4.

The first to be evaluated were selected model cross-sections (Figure 5) to represent the vertical morphological changes in the river bed due to erosion or sedimentation during the 4-year monitoring (Figure 6).

When evaluating the vertical cross-sections, it showed that the changes in the river bed were very heterogeneous. Therefore, we chose the most optimal representation of the river bed changes in the form of envelope curves of the river bed morphology between the first and last year of the monitoring. In each cross-section, we selected one point which details the curves of each monitored year.

If we evaluate the cross-section A-A′ and A′-A′′ (Figure 6(a1)), we may say that the changes occurred up to 2.14 m, and the average change was 0.52 m. It is clear that conditions altered; erosion prevailed in some places, while sedimentation prevailed elsewhere. This was also related to the hydrological conditions in time in the Wisła River, including floods. It is clear from the cross-section A-A′ (Figure 6(a3)) that the most prominent erosion occurred in the first year of monitoring, while later on sedimentation dominated. On the contrary, the cross-section A′-A′′ (Figure 6(a4)) shows the lowest positions in some places in the fourth year of monitoring.

When evaluating the cross-section B-B′ (Figure 6(b1)), the most remarkable changes occurred in the pothole of 180 to 220 m, where the biggest erosion change (2.47 m) was reported in the slope of the hole. In the cross-section C-C’, the biggest changes were observed in the section from 0 to 40 m, and 260 to 280 m on the left slope of the hole. At the elevation at 250 m and at the end of the cross-section behind 400 m (Figure 6(b2)), further significant changes were noted. When evaluating Figure 6(b3), it is clear that there has been a prominent erosion change in the river bed during the monitoring, which correlated with the periods of floods (26 days of enormous water discharge before the third stage of bathymetric measurements (Figure 9a at the top)). The part of the object shown in Figure 6(b4) is significant, since it gives detail information on changes in the morphological elevation, where erosion dominates on the left side and sedimentation on the right.

Erosion processes in the river alluvial geological environment have been studied by [3,24,25,26,27]. It is evident that the formation of potholes is one of the basic hazards for water power plant structures.

### 3.2. Riverbed Capacity/Area Evaluation or Bottom Erosion/Sedimentation Evaluation

**Figure 7:**
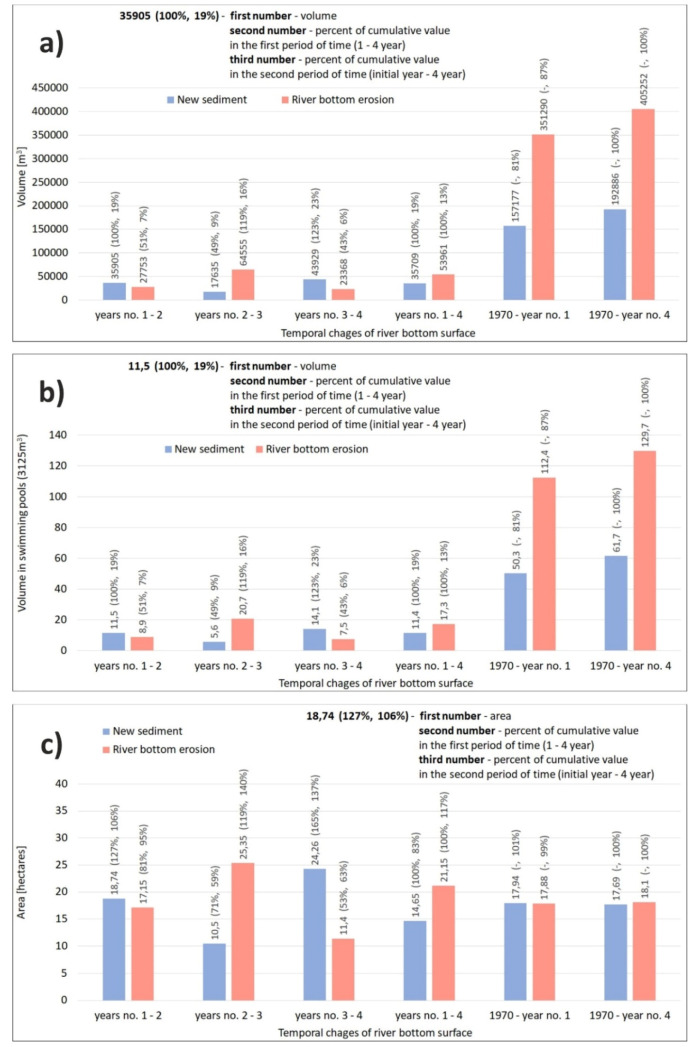
Changes in river bottom erosion and new sediments (**a**) volume (m^3^), (**b**) volume in the number of Olympic-size swimming pools (3125 m^3^), (**c**) area (m^2^).

**Figure 8:**
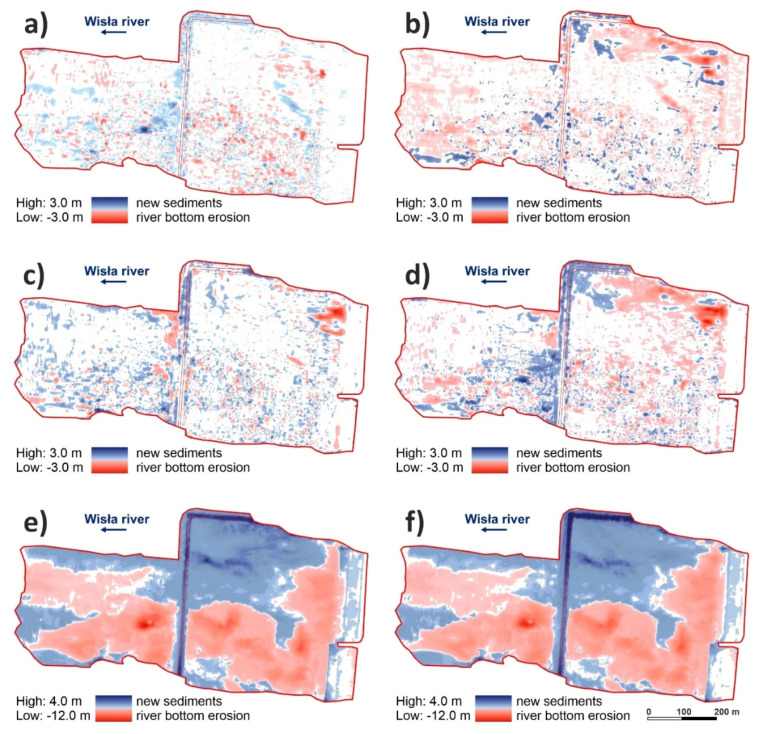
The situation of changes in river bottom surface (river bottom erosion, new sediment), (**a**) between the first and second year of monitoring, (**b**) between the second and third year of monitoring, (**c**) between the third and fourth year of monitoring, (**d**) between the first and fourth year of monitoring, (**e**) between the first year of monitoring and 1970, (**f**) between the fourth year of monitoring and 1970.

When evaluating the changes in the cubic capacity and surface of the Wisła River bed erosion (Figure 7 and Figure 8) during the 4-year monitoring, the most prominent changes occurred between the second and third year, when erosion washed away 64,555 m^3^ of gravel-sand (Figure 7a and Figure 8b). This corresponds to 20.7 Olympic-size pools of 3125 m^3^ (Figure 7b). This amount represents 119% of the final second and third year change. At the same time, it has amounted to 16% of the overall change since the construction to the last year of monitoring. The spatial distribution of the change is shown in Figure 8b. This most prominent change is manifested in the largest area of red colour in Figure 8b (change between year 2 and year 3 of monitoring) when compared to Figure 8a (change between year 1 and year 2 of monitoring), Figure 8c (change between year 3 and year 4 of monitoring), and Figure 8d (change between year 1 and year 4 of monitoring).

During the 4-year monitoring, the final river bed erosion amounted to 53,961 m^3^ (Figure 7a), which is a capacity of 17.3 Olympic-size pools (Figure 7b). However, the 4-year value represents only 13% of the total erosion (Figure 7a) of the river bed since the construction to the end of the monitoring. In other words, the change amounts to 87% of gravel-sand (capacity of 351,290 m^3^, or 112.4 Olympic-size pools (Figure 7b)) since the construction (Figure 7b) to the start of the 4-year monitoring. Therefore, changes were enormous, but distributed in time (Figure 7 and Figure 8).

The most prominent spatial change in erosion (area) was observed between year 2 and year 3, i.e., 25.35 ha (Figure 7c and Figure 8b). During the 4-year monitoring, the area affected by erosion was 21.15 ha (Figure 7c and Figure 8d). Interestingly, the overall spatial change in erosion since the dam construction to the end of monitoring corresponds to a smaller area (18.1 ha) than the area identified during the 4-year monitoring (Figure 7c). This is explained by the fact that the area of interest is too small for the final change to manifest in this parameter during such a long period.

Apart from the erosion process, the geological environment of river alluvia is influenced by sedimentation processes, which was reported in [28,29,30,31,32,33].

When assessing the changes in the cubic capacity of new sediments in the area of interest, we found that the most prominent change during the 4-year monitoring was observed between year 3 and year 4, i.e., 43,929 m^3^ (Figure 7a). This corresponds to 23% of all new sediments since the dam construction. At the end of the 4-year monitoring, the amount was 35,709 m^3^ (Figure 7a, 11.4 Olympic-size pools (Figure 7b)), which is 19% of all new sediments since the dam was constructed. The difference was caused by erosion because the newly settled soils were reduced by intense erosion in year 3 and year 4. The overall change since the dam construction to the end of monitoring was 192,886 m^3^ (Figure 7a), which corresponds to 61.7 Olympic-size pools (Figure 7b).

**Figure 9:**
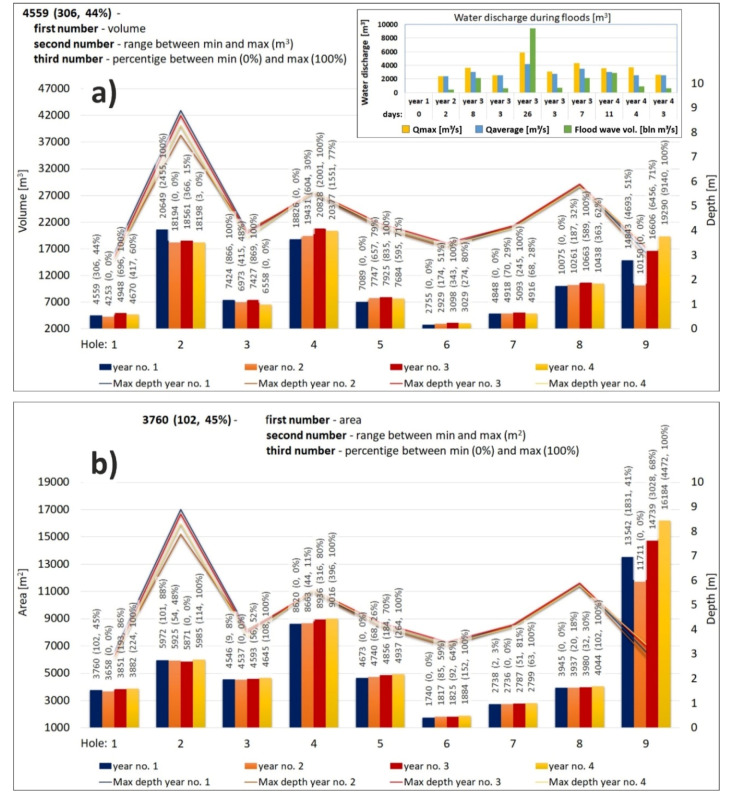
Graph of pothole quantification (**a**) volume (m^3^), (**b**) area (m^2^).

As for the evaluation of new sediments in the area of interest, we found that the biggest quantity of sediments was identified in the area between year 3 and year 4, i.e., 24.26 ha, which corresponds to 137% of the total change since the dam construction (Figure 7c). This may be explained by the biggest flood on the Wisła River being in year 3 (Figure 9a at the top). At the end of the 4-year monitoring, the area affected by new sediments was 14.65 ha, which corresponds to 83% of the total change only. The overall area affected by new sediments is 17.69 ha (Figure 7c).

If we evaluate the changes caused by river bed erosion, as well as the deposition of new sediments related to the geometry of the study area (Figure 8), more significant changes were clearly observed between the year of construction (1970) and the final year of monitoring (Figure 8f) than during the 4-year monitoring (Figure 8d). At the same time, it is possible to observe the dominant direction of pothole formation on the gravel-sand bottom of the Wisła River in the area of interest, i.e., Włocławek dam. This dominant direction is east–west. The direction is an arc, which mirrors the shape of the Wisła River, while it is bound onto the left bank in the direction of water flow. The abundance of potholes is influenced by the location of discharge from the dam on the left bank, and the direction of the dominant flow (east–west) (Figure 5 and Figure 8e,f).

### 3.3. Potholes Evaluation

In the area of interest, there are nine partial potholes (morphological depressions, Figure 2 and Figure 5). The majority of them are in the south of the area of interest, which is caused by the direction of dominant discharge from the dam. On the contrary, the sedimentation is higher in the north.

The graph with pothole quantification is presented in Figure 9. When evaluating the 4-year monitoring of the river bed as for the cubic capacity of the nine measured potholes, the most prominent erosion occurred in pothole 2 and pothole 4 (Figure 9a). The hazard related to the potholes lies in the fact that they are situated in the vicinity of the threshold. In year 3, we identified the capacity of 20,828 m^3^ in pothole 4.

The nine potholes observed in connection with the changes in the cubic capacity were classified into three groups of analogous behaviour (Figure 9). In potholes 1 to 3, which are located behind the threshold, the trend was a gradual increase and decrease in the values during the 4-year monitoring. On the contrary, in potholes 4 to 8, which are situated in front of the threshold in the direction of the dominant flow, there was a rising trend in the first three years of monitoring, followed by a slight decrease in year 4. This is related to the floods in year 3 and year 4 (see Figure 9a at the top). A specific feature is pothole 9, which is located outside the discharge from the dam. Changes manifest here only during floods, i.e., there was a gradual trend of increasing sediments between years 2 and 4.

The examination of the correlations of maximum depths in the different years of monitoring and comparing these to the cubic capacity of the potholes (Figure 9a), renders clear correlations. The only outlier is pothole 9, which is much shallower than the other potholes, which is explained by its location outside the discharge area from the power plant. This means that it gets affected only during floods, when the increased discharge influences this section too.

When evaluating the spatial distribution of the different potholes, it is clear that the largest is pothole 9, i.e., 16,184 m^2^ although its genesis is related to floods and position outside the discharge from the dam. The second largest pothole is pothole 4, with an area of 9016 m^2^ in year 4. Interestingly, this area is less deep than pothole 2, even though pothole 4 is larger. The third largest pothole is pothole 2 (5985 m^2^ in year 4 of monitoring), which is the deepest pothole in the area of interest.

## 4. Customized River Bottom Hazard Map for Remediation Purposes

**Figure 10:**
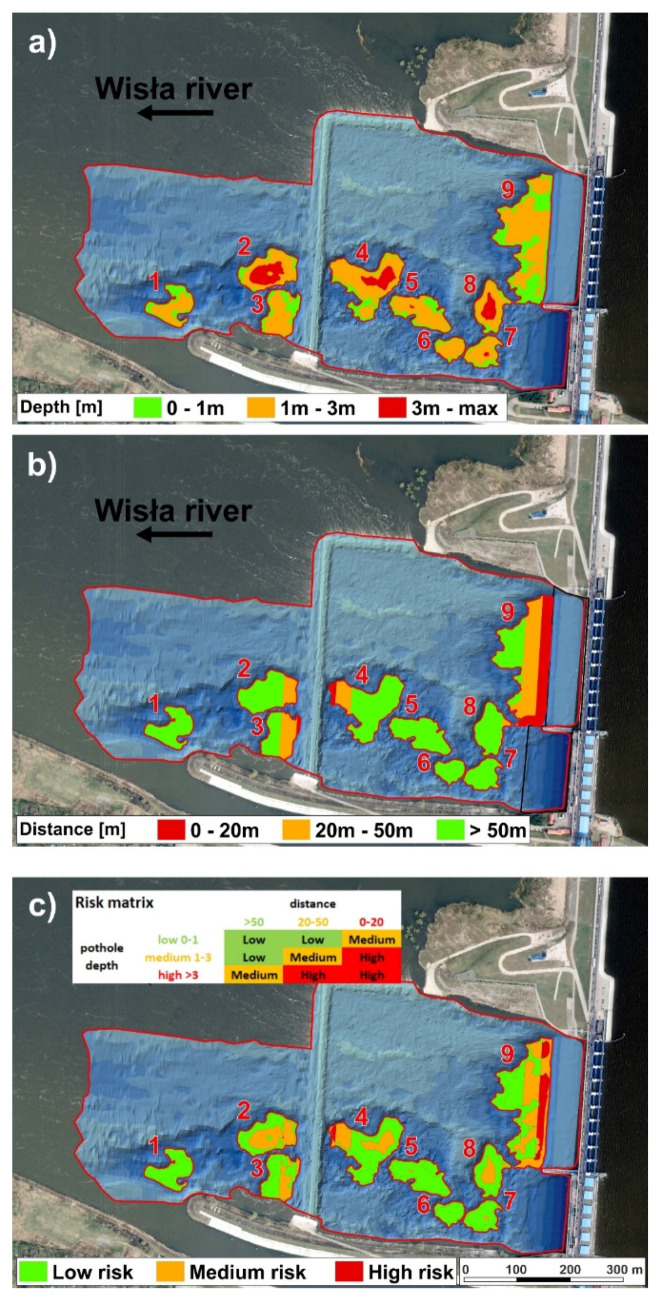
River bottom hazard map (**a**) classification by factor of pothole depth, (**b**) classification by factor of distance from structure of dam and threshold, (**c**) final map classification by combination of these 2 factors (methodology is in risk matrix).

We produced a customized river bottom hazard map for remediation purposes based on the combination of two risk criteria (Figure 10).

The first criterion is the pothole depth (Figure 10a), where the category of low risk is related to potholes 0–1 m based on empirical evidence. The category of medium risk is characterized by pothole depth of 1–3 m. The category of high risk includes potholes over 3 m deep.

The second criterion is the distance from the dam structures (Figure 10b), where, based on empirical evidence, the low-risk category has potholes of more than 50 m from the nearest power plant structure. The distance of potholes from the nearest power plant structures in the medium-risk category is 20–50 m. The potholes in the high-risk category are 0–20 m from the nearest power plant structures.

The final customized river bottom hazard map for remediation purposes was produced via the combination of factors of potholes’ depth and their distance from dam structure. The final categories of low risk, medium risk, and high risk were plotted based on the matrix in Figure 10c.

Figure 10 shows the geometric distribution of both risk factors in the area of interest that influence the final hazard map.

First, we show the first criterion of pothole depth hazard (Figure 10a) distributed in the zones. There we can see that the deepest category of high-risk concerns only some potholes, i.e., potholes 2, 4, 5, 7 and 8.

The second hazard criterion expresses the distance of potholes from the nearest power plant structure, which shows in the map in two ways (Figure 10b). The first is the distance of potholes from the dam, where only a part of pothole 9 is in the most critical zone (0–20 from the dam). In the medium-risk zone, there are parts of potholes 7 and 8. The second distance is the distance of potholes from the threshold, where parts of potholes 3 and 4 are located in the high-risk category, while parts of potholes 2, 3 and 4 are in the medium-risk category.

In the final hazard map (Figure 10c), parts of potholes 4, 9, and a small part of pothole 2 are in the high-risk category. Pothole 9 is located close to the dam, while potholes 4 and 2 are near the threshold. The medium-risk category concerns potholes 2, 3, 4, 5, 7, 8, and 9. Remediation backfilling is recommended only in potholes that combine the high-risk and medium-risk category of pothole depth and are located within 50 m of the power plant structures, i.e., potholes 9, 7, 4, 3, and 2.

**Figure 11:**
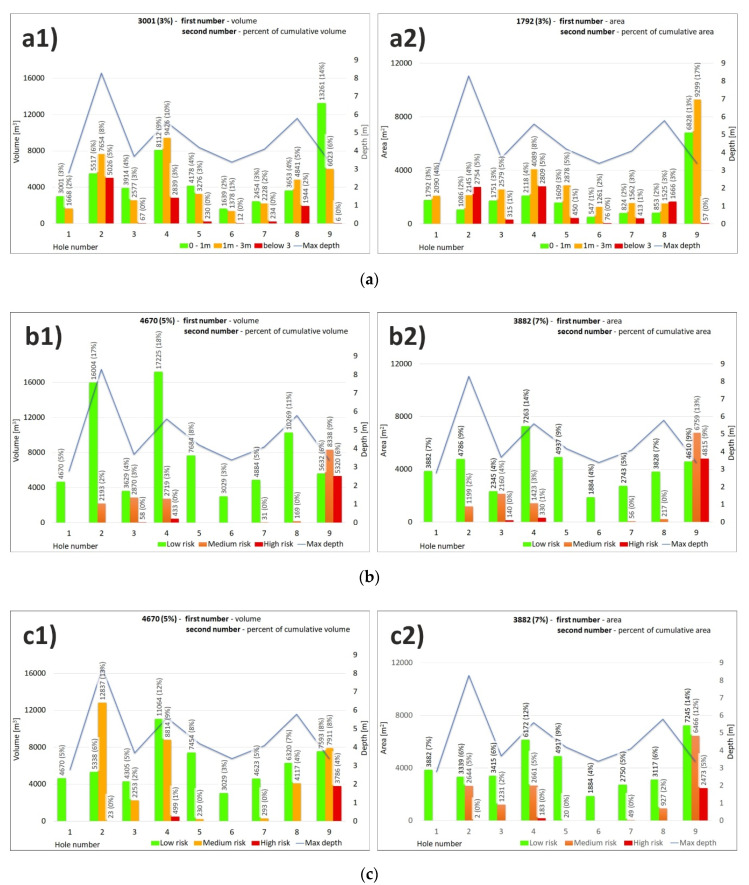
Quantification of risk categories in the special river bottom hazard map for every pothole (**a**) classification by factor of pothole depth, (**b**) classification by factor of distance from structure of dam and threshold, (**c**) final map classification by combination of these two factors: (**a1**,**b1**,**c1**), volume (m^3^), (**a2**,**b2**,**c2**) area (m^2^).

The quantification of the 4-year-monitoring results were plotted as the risk categories in the river bottom hazard map is manifest in Figure 11, while Figure 11(a1,b1,c1) represent the results as cubic capacity (m^3^), and Figure 11(a2,b2,c2) shows the area (m^2^) changes in the categories.

As for the quantification of the first factor (Figure 11(a1,a2)) using the pothole depth, we may say that as many as 7 parts of different potholes were in the high-risk category, while the highest cubic capacity and area are related to parts of pothole 2 (5026 m^3^), 4 (2839 m^3^), and 8 (1944 m^3^). When quantifying the second factor of the pothole distance from the dam/threshold (Figure 11(b1,b2)), the high-risk category includes pothole 9 (5320 m^3^), which is located close to the dam. The least concerning are potholes 4 (433 m^3^) and 3 (58 m^3^). The combined evaluation in the final map of hazard caused a redistribution of the risk categories, while the high-risk category concerns only potholes 9 (3786 m^3^) and 4 (499 m^3^) (Figure 11(c1)).

When looking at the distribution of area from the point of view of the categories, the most abundant is the low-risk category. This is explained by the shape of the potholes, where the largest parts of the potholes are shallow. The second most abundant category is the medium-risk category. The third is thus the high-risk category with the deepest potholes near the power plant structures.

**Figure 12:**
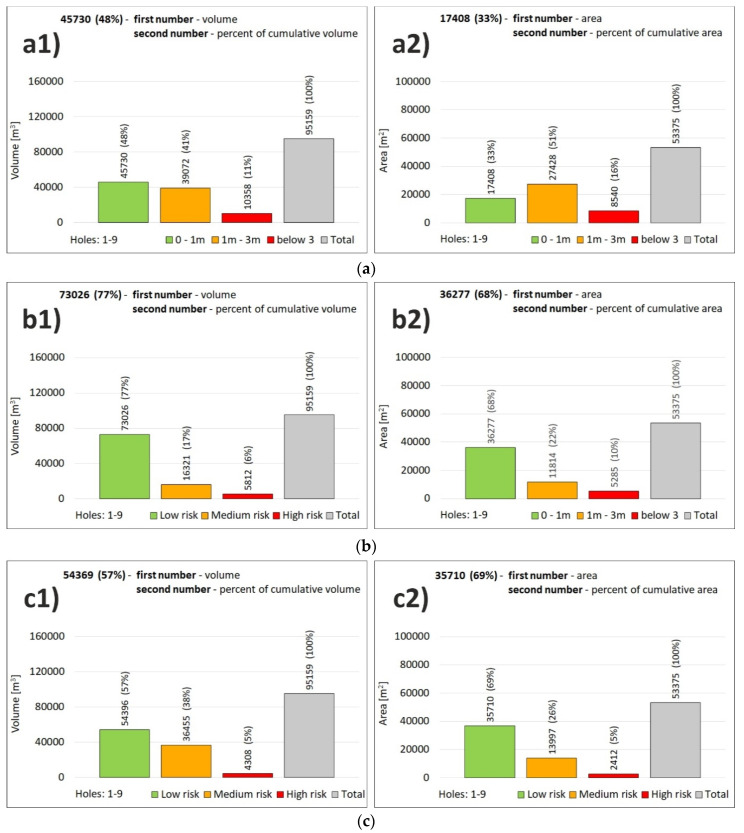
The sum of risk categories in the river bottom hazard map (**a**) classification by factor of pothole depth, (**b**) classification by factor of distance from structure of dam and threshold, (**c**) final map classification by combination of these 2 factors, (**a1**,**b1**,**c1**)—volume (m^3^), (**a2**,**b2**,**c2**)—area (m^2^).

Overall, the capacity of all the potholes amounts to 95,519 m^3^ (Figure 12(c1)) and area of 53,375 m^2^ (Figure 12(c2)). These two characteristics are important for the economic aspects of the remediation process as they express the maximum mass needed to be backfilled. The map in Figure 10 is relevant for the technical implementation, logistics, access roads to the potholes, and distribution of the backfill materials in the area.

The high-risk category is the least abundant with the capacity of 4308 m^3^, which is 5% of all interest area pothole cubic capacity (Figure 12(c1)). As for the area, the value is 2412 m^2^ (Figure 12(c2)). The medium-risk category is the second most abundant with the capacity of 36,455 m^3^ (38% of the total pothole cubic capacity). The area of the category covers 13,997 m^2^, i.e., 26% of all pothole area. The low-risk category is related to pothole capacity of 54,396 m^3^ (57% of the total pothole cubic capacity). As for the area, the category concerns 35,710 m^2^ (69% of all potholes).

## 5. Conclusions

Based on the evaluation of the 4-year engineering-geological geodetic/bathymetric monitoring of Wisła River bottom in the area of Włocławek water power plant, we may conclude that there is a dynamic erosion activity, which leads to the formation of potholes. It is evident that the distribution of the activity is heterogeneous in time and space, while there is a dominant east–west direction in the formation caused by the location of the discharge from the dam near the left bank. The southern part of the river bed is clearly influenced by the formation of potholes due to erosion. On the other hand, the north side is rather characterized by increased sedimentation.

The quantification of these changes shows that the most prominent changes occurred between 1970 (year of dam construction) and the beginning of the 4-year monitoring, i.e., capacity of 351,290 m^3^ (87%). The 4-year monitoring covered only 13% of the total loss in the alluvial sediments due to erosion, and only a 19% increase in the new sediments. This implies that it is not important to carry out an annual monitoring of the whole area, but focus on the zones near the dam and threshold only.

Using a new methodology, we produced a special customized river bottom hazard map for remediation purposes, which considered two risk factors, i.e., the pothole depth and the pothole distance from the power plant structures. As for the risk categories, the high-risk category contains 5% of potholes (4308 m^3^), the medium-risk category contains 38% of potholes (36,455 m^3^), and the low-risk category has 57% of potholes (54,396 m^3^). The overall pothole capacity is 95,159 m^3^. The newly proposed methodology for pothole assessment is useful for any dam in analogous geological conditions, i.e., with alluvial gravel-sand sediments in the surroundings of dam structures.

The research resulted in a remediation project plan in the form of pothole backfilling. We proposed to backfill only those potholes that combine the high-risk and medium-risk category with the distance below 50 m from the power plant structures. The 4-year monitoring helped us to understand the spatial and depth heterogeneity of pothole occurrence to be marked in a map, based on which backfilling may be implemented. The 4-year long monitoring showed there is no need for annual monitoring. It is sufficient to carry out monitoring biannually, and the monitoring may be selective only with the high-risk zone within 20 m from the power plant concrete structures.

The methodology of the research study may be applied to other water structures with analogous conditions and problems. In case of such problems, it is important to deal with the pothole remediation, as well as to design optimal distances between water structures, in order to ensure an optimal water flow stabilization.

## Figures and Tables

**Table 1:** Equipment used during bathymetric measurements.

	Equipment	GPS	GNSS	SBES	RTS
Parameters					
Manufacturer Model		Ashtech Z-Xtreme	Topcon Hiper Pro	Simrad EA501P	Leica Nova MS50
Measurement method		GPS RTK positioning	GNSS OTF positioning	Depth sounding 200 kHz	Total station positioning
Declared accuracy		H: 0.02 m/V: 0.05 m	H: 0.02 m/V: 0.03 m	0.01 m	Hz/V: 1′′/3^cc^ D: 1 mm + 1.5 ppm
Calibration method		Control point	Control point	Sound Velocity Profiler Bar Check Calibration	Control point
Effective accuracy		H: 0.02 m/V: 0.02 m	H: 0.01 m/V: 0.02 m	0.02 m	H: 0.02 m/V: 0.04 m 400 m from base station
